# New strategy of early surgery for infective endocarditis complicated by intracranial hemorrhage

**DOI:** 10.1007/s00595-024-02964-1

**Published:** 2024-11-27

**Authors:** Shota Hasegawa, Hiroaki Takahashi, Katsuhiro Yamanaka, Kenji Okada

**Affiliations:** Division of Cardiovascular Surgery, Department of Surgery, Kobe University Graduate School of Medicine, 2-5-7, Kusunoki-cho, Chuo-ku, Kobe, 650-0017 Japan

**Keywords:** Intracranial hemorrhage, Infective endocarditis, Anticoagulant, Nafamostat mesilate

## Abstract

**Purpose:**

Early surgery for infective endocarditis with intracranial hemorrhage can cause severe bleeding, which is correlated with an increased mortality. In 2005, we started using nafamostat mesilate and low-dose heparin as anticoagulants during cardiopulmonary bypass for early surgery. The outcomes of this strategy have been reviewed.

**Methods:**

All patients who underwent cardiac surgery for active infective endocarditis with intracranial hemorrhage between 2005 and 2023 were evaluated.

**Results:**

There were 23 consecutive patients (median age 62 years old). Ten patients (43%) had neurologic deficits. The indication for early surgery in most patients was the presence of mobile vegetation or existing embolic events (18 of 23, 78%). No complications were associated with cardiopulmonary bypass. The median interval between the diagnosis and surgery was two days. There was 1 early death (4%) due to sepsis. There was no exacerbation of intracranial hemorrhage. One patient had new ectopic microbleeds without deterioration of neurologic findings. One patient had a new-onset cerebral infarction with neurologic deficits. None of the patients exhibited neurologic deterioration. The median follow-up duration was 26 months. overall survival was 90.7% after 5 years.

**Conclusions:**

Our strategy of using nafamostat mesilate enabled us to safely perform early surgery in patients with intracranial hemorrhage without hemorrhage exacerbation.

## Introduction

While guideline recommendations currently favor early surgery for infective endocarditis (IE) if complications develop during medical treatment [1, 2], the optimal timing of surgery is still controversial in patients with cerebral complications. Intracranial hemorrhage is the most serious complication, as high-dose systemic anticoagulation during cardiac surgery can exacerbate bleeding and lead to increased mortality [3, 4].

A multicenter cohort study demonstrated that early surgery for patients with intracranial hemorrhage may cause new bleeding, neurologic deterioration, and a poor prognosis [5]. Based on this, the American Heart Association/American College of Cardiology 2017 Guidelines recommend delayed surgery, defined as at least 4 weeks, for patients with major ischemic stroke or intracranial hemorrhage [6]. However, some patients occasionally require early surgery due to progressive heart failure, uncontrolled infection, or recurrent embolic events.

To solve this dilemma and accomplish early surgery for patients with intracranial hemorrhage while minimizing the risk of further bleeding, we have used nafamostat mesilate (NM; 6-amino-2 naphthalene-p-guanidinobenzoate dimethanesulfonate) and low-dose heparin as anticoagulants during cardiopulmonary bypass since 2005, as our group has reported previously [7–10]. Our group demonstrated the usefulness of NM with its very short half-life and ability to inactivate coagulation, fibrinolysis, and platelet aggregation.

Previous studies have reported the outcomes of this innovative strategy in patients with acute stroke, which were not limited to cases with intracranial hemorrhage. In the present study, we reviewed our strategy and experience, focusing specifically on the most challenging patients with IE complicated by intracranial hemorrhage.

## Methods

### Indication for surgical timing

In cases of intracranial hemorrhage, our ideal policy is to pursue non-surgical management for approximately one month if possible. However, in a practical clinical setting, if there is uncontrolled infection, heart failure, or a high risk of recurrent embolization, and there is no severely decreased level of consciousness, we take an aggressive approach to early surgery, regardless of the amount of bleeding.

### Study design

This retrospective study evaluated all patients with active IE and intracranial hemorrhage who underwent cardiac surgery with cardiopulmonary bypass using low-dose heparin and NM at Kobe University Graduate School of Medicine between June 2005 and February 2023. Intracranial hemorrhage includes a spectrum of diseases, ranging from incidental microbleeds, ischemic stroke with hemorrhagic transformation, subarachnoid hemorrhage, and parenchymal hemorrhage to more significant lesions. All patients were diagnosed with active IE according to the modified Duke criteria.

Intracranial hemorrhage was detected using magnetic resonance imaging (MRI) or computed tomography (CT) preoperatively. At the same time, patients were also assessed for the presence of a cerebral aneurysm using magnetic resonance angiography or CT angiography. If it was considered the cause of the hemorrhage or if the risk of rupture was considered high, intervention of the aneurysm was performed by a neurosurgeon, with the intervention method determined by the neurosurgeon.

Postoperative imaging tests were performed within a day after cardiac surgery to monitor any worsening neurologic findings in all patients. Medical records were reviewed, and follow-up was performed by reviewing outpatient charts or by contacting the referring physicians.

The institutional ethics committee approved the anonymous use of patient data (approval 2022-0098; approved on November 9, 2021), and the need for individual consent was waived.

### Anticoagulation protocol during cardiopulmonary bypass (CPB)

In 2005, we started using nafamostat mesylate was used as an anticoagulant during CPB in patients with active IE complicated by cerebral complications [7]. Before cannulation for CPB, 100 IU/kg of heparin (a third of the normal dosage) was administered to achieve an activated clotting time (ACT; measured by a Hemochron^TM^; Accriva Diagnostics, Inc., San Diego, CA, USA) of over 200 s. After this was accomplished, cannulae were inserted. If the ACT did not increase beyond 400 s, 20 mg of NM or 500–1000 IU of heparin was administered.

**Fig. 1:**
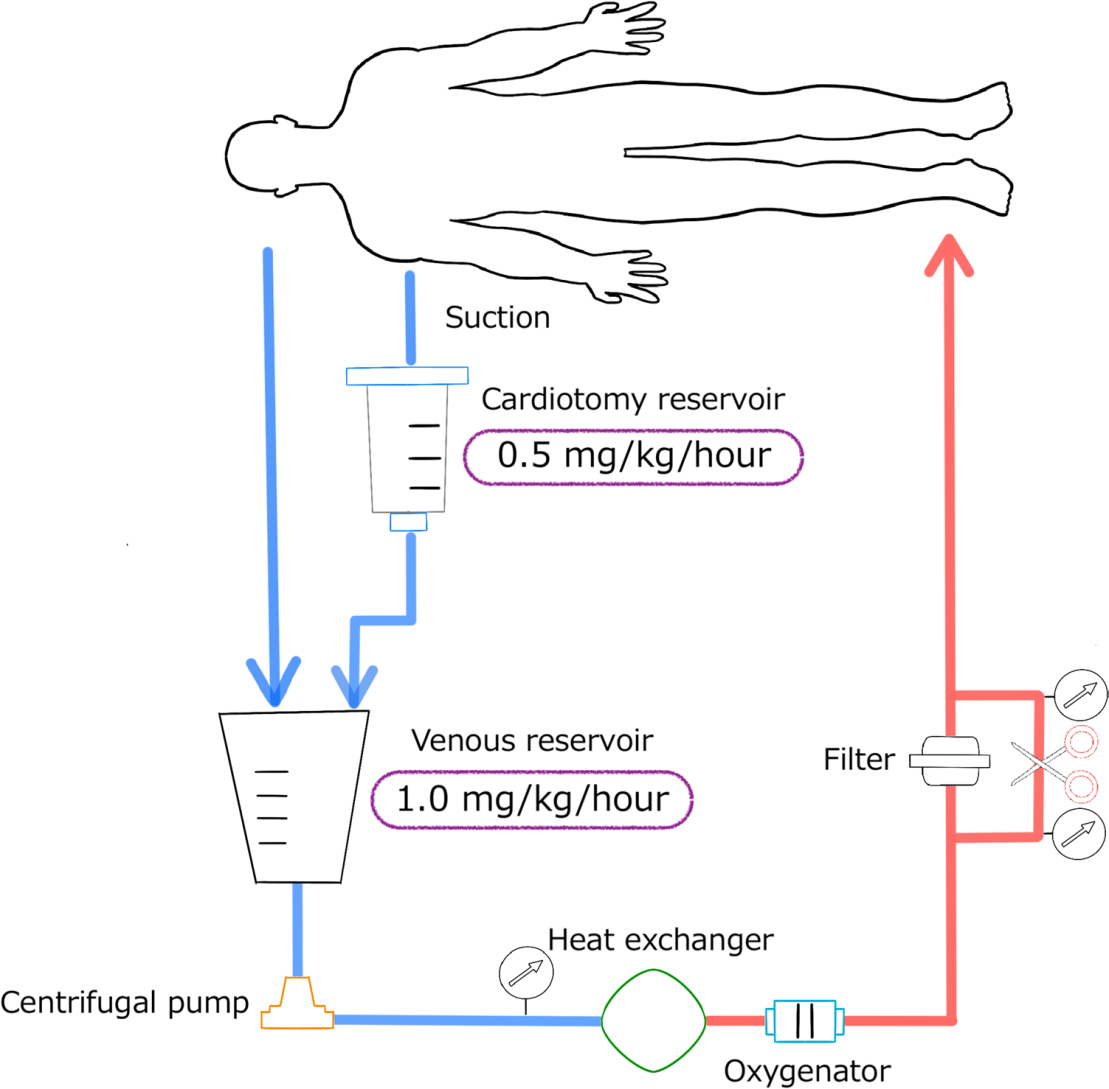
Schema of the pump circuit. Nafamostat mesilate was infused continuously into the cardiotomy reservoir and venous reservoir at 0.5 and 1.0 mg/kg/h, respectively. The dosage of injection changed according to activated clotting time

The pump circuit was composed of a centrifugal pump, silicon- and heparin-coated hollow-fiber polypropylene oxygenator, and heparin-coated bypass circuit (Fig. 1). After connecting to the circuit, the NM was infused continuously through the venous line of the pump circuit at 1.0 mg/kg/h. The ACT was maintained at 300–600 s by a fine titration of the NM dose every 15 min, which was also added at a constant rate (0.5 mg/kg/h) to the cardiotomy reservoir. The NM dose to the venous reservoir was adjusted to maintain the ACT at its optimal level. There was no upper limit for the NM dose. An additional bolus injection of heparin was administered if the ACT decreased to <200 s or in the presence of visible viscosity of the blood collected in the pericardial cavity. the patient’s core temperature was reduced to 33 ℃, and the target mean systemic pressure was 50 mmHg.

After weaning from CPB and decannulation, no pump suction was allowed, and protamine was not usually administered, except when excess heparin was noted after the ACT assay.

The study protocol was approved by the ethics committee of our institution.

### Study outcomes

The primary outcomes were the survival and neurologic deterioration. Early death was defined as death during the hospital stay or within 30 days after surgery. Neurologic deterioration was defined as the worsening of neurologic findings due to new-onset stroke or hemorrhage.

The secondary outcome was new-onset intracranial hemorrhage or exacerbation of intracranial hemorrhage, which was diagnosed using imaging tests.

### Statistic analysis

Descriptive statistics were described as absolute numbers and percentages for categorical variables. Continuous variables are expressed as mean values with standard deviations or median values with range. The overall survival was estimated using the Kaplan–Meier method. All statistical calculations were performed using the JMP Pro software program, ver. 15.0.0 (SAS Institute, Cary, NC, USA).

## Results

### Patient population

A total of 23 patients were enrolled in this study. Patient characteristics are shown in Tables 1 and 2.

**Table 1:** Patient characteristics

Variables	*n* = 23
Age, yrs	56.6 ± 18
Male, *n* (%)	14 (61)
Diabetes mellitus, *n* (%)	4 (17)
Hypertension, *n* (%)	7 (30)
Peripheral artery disease, *n* (%)	0
Hemodialysis, *n* (%)	1 (4)
Atopic dermatitis, *n* (%)	3 (13)
Steroid use, *n* (%)	2 (9)
Old stroke, *n* (%)	1 (4)
History of cardiac surgery, *n* (%)	7 (30)
Atrial fibrillation, *n* (%)	2 (9)
Anticoagulation, *n* (%)	3 (13)
Antiplatelet agent, *n* (%)	4 (17)
Left ventricular ejection fraction <50 %, *n* (%)	0
NYHA class ≥III, *n* (%)	10 (44)
Valvular disease severity ≥moderate, *n* (%)	17 (74)
Shock, *n* (%)	5 (22)
Intubation, *n* (%)	6 (26)
Japan SCORE II, %	19.5 ± 19
Type of endocarditis, *n* (%)	
Native valve	16 (70)
Native valve, reoperation	3 (13)
Prosthetic valve	4 (17)
Vegetation site, *n* (%)	
AV	5 (22)
MV	15 (65)
AV + MV	3 (13)
Microbiologic findings, *n* (%)	
* Staphylococcus aureus*	10 (44)
Viridans *Streptococci*	10 (44)
* Enterococcus faecalis*	2 (9)
* Corynebacterium*	1 (4)

*AV* aortic valve, *MV* mitral valve, *NYHA* New York Heart Association

**Table 2:** Preoperative status, imaging findings, and indication of surgery

Variables	*n* = 23
Neurologic findings, *n* (%)	
Hemiplegia	5 (22)
Disordered consciousness	2 (9)
Aphasia	5 (22)
Blindness	1 (4)
No deficit	14 (61)
Sub-arachnoid hemorrhage, *n* (%)	6 (26)
Hemorrhagic lesion, *n* (%)	
<10 mm	8 (35)
≥10 mm, <30 mm	6 (26)
≥30 mm	3 (13)
Rupture of mycotic aneurysms, *n* (%)	4 (17)
Time to surgery from diagnosis, days	
Median (range)	2 [1–11]
1–7 days, *n* (%)	20 (87)
8–14 days, *n* (%)	3 (13)
≥15 days, *n* (%)	0
Indication for early surgery, *n* (%)	
Mobile vegetation/emboli	18 (78)
Uncontrolled infection	9 (39)
Uncontrolled congestive heart failure	4 (17)

**Fig. 2:**
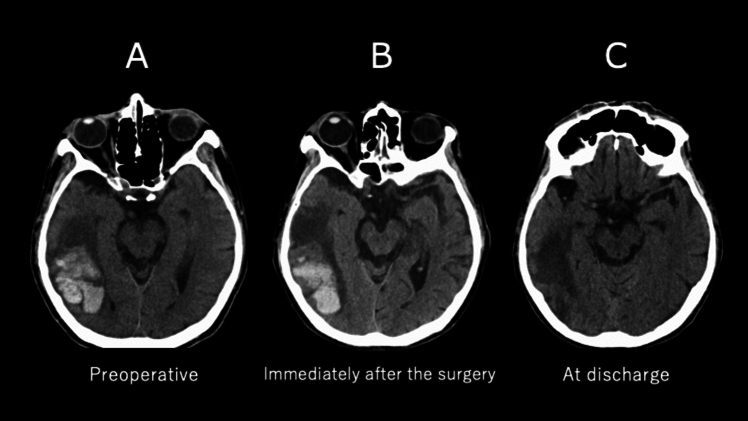
Computed tomographic images of a case with a large hemorrhagic lesion. A 62-year-old male had a hemorrhagic infarction in his right temporal lobe. He underwent a mitral valve replacement 6 days after the onset of the hemorrhagic infarction, and no enlargement of the hemorrhagic lesion was observed. **A** Preoperative image. **B** An image immediately after surgery. **C** An image at discharge

Six patients were intubated prior to arrival in the operating room: four had congestive heart failure and two had cardiogenic or septic shock. One patient with cardiogenic shock required a preoperative intra-aortic balloon pump insertion. Four patients had prosthetic valve endocarditis, and all of them had undergone aortic valve replacement. Three patients had preoperative hemorrhagic sites >30 mm in size (Table 2, Fig. 2). One patient underwent trapping of a ruptured intracranial mycotic aneurysm 8 days before cardiac surgery, and three patients underwent embolization of a ruptured aneurysm with n-butyl-2-cyanoacrylate 1 or 3 days before cardiac surgery.

Follow-up was 100% complete, with a median follow-up time of 26 months (interquartile range, 4–75).

### Operative data

**Fig. 3:**
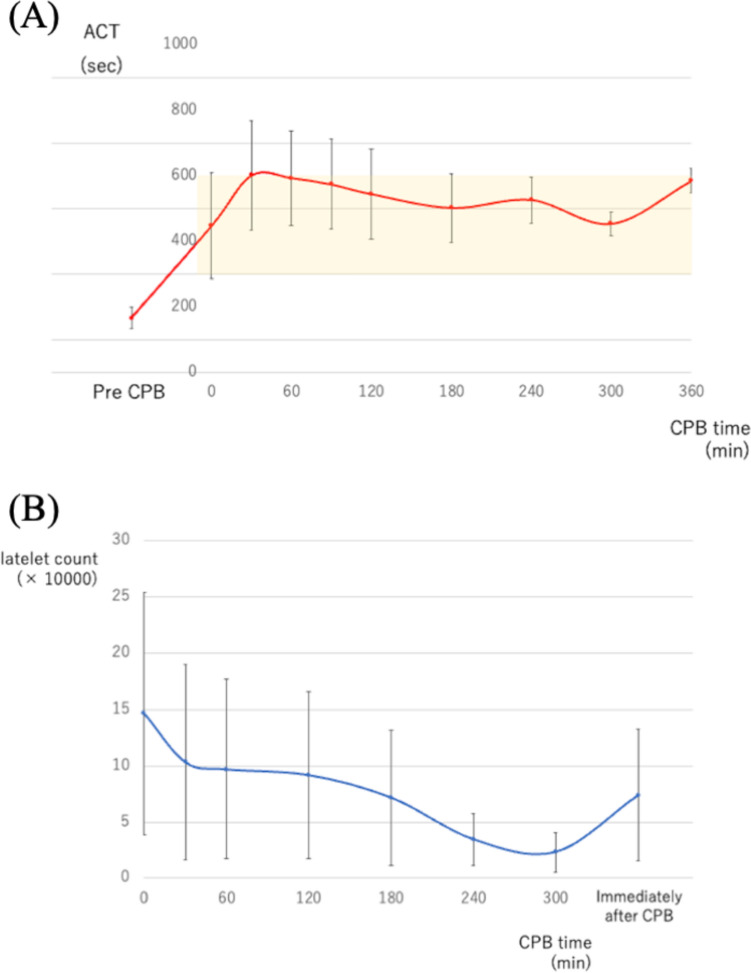
**A** Changes in mean ACT values during surgery with CPB time. Error bars indicate one standard deviation of the mean. The yellow area represents the target range. (*ACT* activated clotting time, *CPB* cardiopulmonary bypass) **B** Changes in mean platelet count during surgery. Error bars indicate one standard deviation of the mean. (*CPB* cardiopulmonary bypass)

Operative details are presented in Table 3. No patient experienced side effects such as agranulocytosis, hyperkalemia, or anaphylactic reactions caused by nafamostat mesilate. The median cumulative dose of nafamostat mesilate was 276.5 (108–683) mg. The mean cumulative heparin dosage in patients who underwent cardiopulmonary bypass using nafamostat mesilate was 149 IU/kg, while the median was 109.0 (46.0–266.7) IU/kg. The ACT was controlled within the targeted range during CPB and normalized immediately after CPB termination without protamine in all patients (Figure 3A).

**Table 3:** Operative data

Variables	*n* = 23
Cardiopulmonary bypass time, min	176 [84–525]
Aortic cross-clamp time, min	135 [56–430]
MV repair, *n* (%)	3 (13)
MV replacement, *n* (%)	11 (48)
Biologic, *n* (%)	10 (43)
Mechanical, *n* (%)	1 (4)
AV repair, *n* (%)	0
AV replacement, *n* (%)	4 (17)
Biologic, *n* (%)	4 (17)
Mechanical, *n* (%)	0
TV replacement, *n* (%)	1 (4)
Biologic, *n* (%)	1 (4)
Aortic root replacement, *n* (%)	3 (13)
Bio-Bentall, *n* (%)	2 (9)
Mechanical-Bentall, *n* (%)	1 (4)
Commando operation, *n* (%)	2 (9)

*AV* aortic valve, *MV* mitral valve, *TV* tricuspid valve

### CPB management

No complications were associated with CPB. The mean maximum pressure at the pre-oxygenator was 162.8 ± 31 mmHg, while the mean arterial pressure was 50 mmHg during CPB. The mean minimum rectal temperature was 32.6 ± 0.9 °C. The changes in ACT and platelet counts during CPB are shown in Fig. 3.

### Primary outcomes

The clinical outcomes are summarized in Table 4. There was one case of early death. She died of sepsis three months after mitral valve replacement due to recurrent infection.

**Table 4:** Clinical endpoints

Variables	*n* = 23
In-hospital death, *n* (%)	1 (4)
Neurologic deterioration, *n* (%)	1 (4)
Exacerbation of hemorrhage, *n* (%)	0
New intracranial hemorrhage, *n* (%)	1 (4)
New cerebral infarction, *n* (%)	1 (4)
Rupture of mycotic aneurysm, *n* (%)	2 (9)
Systemic embolization, *n* (%)	0
Re-exploration for bleeding, *n* (%)	0
ICU stay, days, median [range]	5 [2–91]
Dosage of nafamostat mesilate, mg	288.7 ± 137

There was one late death, wherein the patient died of acute heart failure due to sick sinus syndrome three months after the surgery. The overall survival was 90.7% ± 1.3% at 5 and 10 years.

There was one case of neurologic deterioration; this patient, without preoperative neurologic deficits, had a new onset of cerebral infarction in the left middle cerebral artery territory after the Commando operation. He suffered from aphasia and right hemiplegia without aggravation of intracranial hemorrhage. The patient had no CPB-associated complications.

### Secondary outcomes

**Fig. 4:**
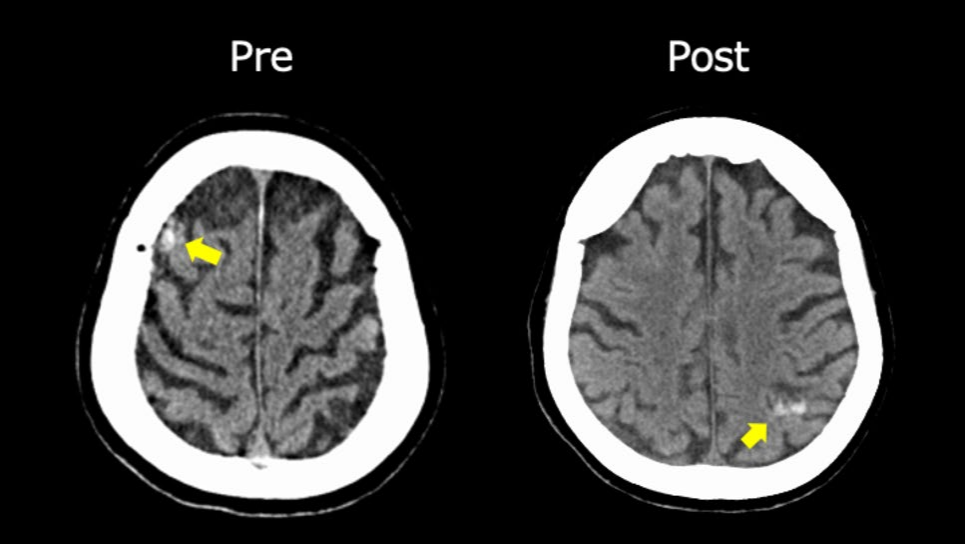
Pre- and postoperative computed tomographic images of patients with postoperative microbleeds. A 83-year-old female had an ectopic microbleeds after valve surgery using nafamostat mesilate. *Yellow arrows* indicate hemorrhagic lesions

There was no exacerbation of intracranial hemorrhage. One patient had new ectopic cerebral microbleeds on imaging with no worsening of neurologic findings (Fig. 4). This patient had an ischemic stroke with hemorrhagic transformation, and the preoperative lesion size was 9 mm. She underwent mitral valve replacement using nafamostat mesilate, and microbleeds were found at another site. New bleeding was found on a screening test with CT on the day after surgery. The patient showed no changes in known preoperative hemorrhagic sites. In this case, the CPB time was 176 min, and the ACT did not clearly deviate from the target range during surgery.

## Discussion

In patients with active IE, the timing of surgery should be decided based on clinical manifestations, including uncontrollable infection, progressive heart failure, or systemic embolism. A randomized trial demonstrated that early surgery significantly reduced the risk of death and embolic events compared with conventional therapy [11]. However, in patients with intracranial hemorrhage, early surgery has been reported to result in an increased incidence of in-hospital death related to new severe bleeding after cardiac surgery [5, 12]. Indeed, a multicenter observational study reported that patients with intracranial hemorrhage who underwent surgery within 4 weeks of a hemorrhagic event had a higher mortality than those who underwent surgery after more than 4 weeks (75% versus 40%, respectively) [5]. The percentage of new bleeds postoperatively was reported to be 50% in surgeries performed in the first 2 weeks after the neurologic event.

Based on these findings, the American Association for Thoracic Surgery consensus guidelines recommend delaying surgery for 1–2 weeks in patients with non-hemorrhagic stroke and for 3–4 weeks in patients with hemorrhagic stroke to reduce the risk of further intracranial bleeding during heart surgery (Class IIa recommendation, level of evidence B). The 2017 American Heart Association/American College of Cardiology guidelines corroborate this recommendation (Class IIb recommendation) [1].

However, these recommendations are not always applicable to all patients. The timing of surgery should be individualized and determined by weighing the need for surgery against the risk of additional emboli during the waiting period or provoking intracranial hemorrhage during the operation. According to the European Society of Cardiology 2023 guidelines for the management of IE, urgent or emergency surgery should be considered in patients with ICH and unstable clinical status due to heart failure, uncontrolled infection, or persistent high embolic risk, weighing the likelihood of a meaningful neurologic outcome [2]. We believe that if new bleeding can be controlled in patients with intracranial hemorrhage, more patients could benefit from early surgery.

In the present study, 87% of patients underwent surgery within 7 days of the diagnosis with IE and intracranial hemorrhage. Although 4% of the patients had new ectopic microbleeds, none of the patients experienced exacerbation of bleeding or worsening neurologic findings as a result. Few reports have analyzed patients with intracranial hemorrhage who underwent surgery for IE, especially when surgery was performed early. The reported rates of in-hospital mortality in these patients range from 4 to 75% with either early or late interventions [3, 5, 12–15]. Causes of mortality included deaths related to new bleeding events. The mortality rate in the present study was lower than that in previous studies, despite early surgery being performed, perhaps because of the suppression of deaths related to the exacerbation of hemorrhage. An additional reason is that 35% of the patients in this series had microbleeds preoperatively. Sorabella et al. reported that, in patients with microbleeds, early surgical intervention did not lead to significantly worse postoperative outcomes [16]. Several cases of early surgery in patients with large hemorrhagic lesions have been reported to carry a higher risk of exacerbation. However, bleeding was not exacerbated in present study. Therefore, we believe that our strategy is safe and effective and should be considered the treatment of choice for patients with intracranial hemorrhage. Nevertheless, further studies are required to investigate the surgical outcomes in patients with large hemorrhagic lesions.

Nafamostat mesilate is a synthetic low-molecular-weight serine protease inhibitor with a very short half-life that inhibits coagulation and platelet aggregation by deactivating the action of plasmin, thrombin, and activated coagulation factors as well as interfering with fibrinolysis [17]. In the case of high-risk bleeding, nafamostat mesilate has been used as an alternative anticoagulant to heparin in mechanical circulatory support devices and hemodialysis, as it acts as a regional anticoagulant in extracorporeal circuits. Murase et al. proved that the use of nafamostat mesilate during CPB reduced postoperative blood loss without loss of platelet count or function [18]. As their analysis focused on elective CABG patients, the CPB duration was relatively short. In our series, a gradual decrease in the platelet count was observed during CPB, particularly after 4 h. Therefore, we believe that caution is needed for the potential worsening of bleeding in surgeries that require CPB for over 4 h to complete. Fortunately, none of the patients in this study required re-exploration for postoperative bleeding, although there were cases with relatively long CPB times.

Regarding the monitoring of anticoagulation, it has been reported that both ACT and activated partial thromboplastin time are often used in extracorporeal membrane oxygenation management [19]. However, ACT has been used for anticoagulation monitoring because of the need for frequent measurements and fine-tuning of anticoagulant dosing. Whether or not the effects of nafamostat mesilate can be determined solely by monitoring ACT is controversial. During surgery, it is crucial to closely communicate with the perfusionist to assess the actual blood viscosity in the surgical field and adjust the nafamostat mesilate dosage accordingly in relation to the ACT values. In the present study, there were no problems with the CPB circuit, including cases of abnormally high pressure in the preoxygenator. Unfortunately, one patient experienced cerebral infarction postoperatively, the cause of which could not be determined. In addition, there is a future prospect of considering the use of thromboelastography to monitor the anticoagulant effects of NM.

Our group previously reported the results of early surgery in patients with acute stroke [7–10]. Without changing our strategy from previous studies, we focused on cases of intracranial hemorrhage alone in the present study, which requires the most difficult decisions. In addition, cases with longer CPB times were included, including those requiring extensive debridement and reconstruction, such as the Commando procedure. As CPB time increases, there is a concern that the risk of exacerbated intracranial bleeding may increase due to platelet consumption, impaired platelet function, and excessive fibrinolysis. However, even with the inclusion of very high-risk cases, none of the patients showed worsening neurologic findings. These results also demonstrated the safety and efficacy of this strategy using nafamostat mesilate.

### Limitations

The study design has limitations inherent to a single-center retrospective follow-up study that enrolled a small number of patients. We had a small number of patients, and approximately 30% were followed up for longer than 5 years. Because the hemorrhagic lesion in approximately half of the cases was <10 mm in size, this strategy is not considered applicable to all patients with intracranial hemorrhage. Since the routine imaging test in the postoperative acute phase was performed with CT, it is possible to underestimate cerebral events. Furthermore, it is possible that some patients with severe cerebral hemorrhage were either not referred by cardiologists or emergency physicians or that their families declined cardiac surgery during the study period.

## Conclusions

Early surgery using NM in patients with active IE and intracranial hemorrhage can be performed without hemorrhage exacerbation in unavoidable instances.

## References

[1] Pettersson GB, Coselli JS, Hussain ST, Griffin B, Blackstone EH, Gordon SM, et al. 2016 The American Association for Thoracic Surgery (AATS) consensus guidelines: surgical treatment of infective endocarditis: executive summary. J Thorac Caridiothorac Surg. 2017;153:1241–58. 10.1016/j.jtcvs.2016.09.093 28365016

[2] Delgado V, Ajmone Marsan N, de Waha S, Bonaros N, Brida M, Burri H, et al. 2023 ESC Guidelines for the management of endocarditis: Developed by the task force on the management of endocarditis of the European Society of Cardiology (ESC) Endorsed by the European Association for Cardio-Thoracic Surgery (EACTS) and the European Association of Nuclear Medicine (EANM). European heart journal. 2023;44:3948–4042. 37622656 10.1093/eurheartj/ehad193

[3] Diab M, Guenther A, Scheffel P, Sponholz C, Lehmann T, Hedderich J, et al. Can radiological characteristics of preoperative cerebral lesions predict postoperative intracranial haemorrhage in endocarditis patients? Eur J Cardiothorac Surg. 2016;49:e119–26. 26888461 10.1093/ejcts/ezw014

[4] Derex L, Bonnefoi E, Delahaye F. Impact of stroke on therapeutic decision making in infective endocarditis. J Neurol. 2010;257:315–21. 19876684 10.1007/s00415-009-5364-3

[5] García-Cabrera E, Fernández-Hidalgo N, Almirante B, Ivanova-Georgieva R, Noureddine M, Plata A, et al. Neurological complications of infective endocarditis risk factors, outcome, and impact of cardiac surgery: a multicenter observational study. Circulation. 2013;127:2272–84. 23648777 10.1161/CIRCULATIONAHA.112.000813

[6] Nishimura RA, Otto CM, Bonow RO, Carabello BA, Erwin JP, Fleisher LA, et al. 2017 AHA/ACC focused update of the 2014 AHA/ACC guideline for the management of patients with valvular heart disease. J Am Coll Cardiol. 2017;70:252–89. 28315732 10.1016/j.jacc.2017.03.011

[7] Sakamoto T, Kano H, Miyahara S, Inoue T, Izawa N, Gotake Y, et al. Efficacy of nafamostat mesilate as anticoagulation during cardiopulmonary bypass for early surgery in patients with active infective endocarditis complicated by stroke. J Heart Valve Dis. 2014;23:744–51. 25790622

[8] Ota T, Okada K, Kano H, Okita Y. Cardiopulmonary bypass using nafamostat mesilate for patients with infective endocarditis and recent intracranial hemorrhage. Interact Cardiovasc Thoracic Surg. 2007;6:270–3. 10.1510/icvts.2006.146209 17669840

[9] Morimoto N, Henmi S, Yoshida M, Mukohara N. Cardiopulmonary byass strategy with low-dose heparin and nafamostat mesilate in cardiac surgery: a safe option for patients with acute stroke. J Thorac Cardiovasc Surg. 2012;144:726–8. 22541513 10.1016/j.jtcvs.2012.03.030

[10] Yamazato T, Oyama N, Fujii T, Abe N, Ikemiya Y, Tamashiro Y, et al. Aggressive early surgical strategy in patients with intracranial hemorrhage: a new cardiopulmonary bypass option. Gen Thorac Cardiovasc Surg. 2022;70:602–10. 34813003 10.1007/s11748-021-01743-w

[11] Kang DH, Kim YJ, Kim SH, Sun BJ, Kim DH, Yun SC, et al. Early surgery versus conventional treatment for infective endocarditis. N Engl J Med. 2012;366:2466–73. 22738096 10.1056/NEJMoa1112843

[12] Okita Y, Minakata K, Yasuno S, Uozumi R, Sato T, Ueshima K, et al. Optimal timing of surgery for active infective endocarditis with cerebral complications: a Japanese multicentre study. Eur J Cardiothorac Surg. 2016;50:374–82. 26968761 10.1093/ejcts/ezw035

[13] Yoshioka D, Toda K, Sakaguchi T, Okazaki S, Yamauchi T, Miyagawa S, et al. Valve surgery in active endocarditis patients complicated by intracranial haemorrhage: the influence of the timing of surgery on neurological outcomes. Eur J Cardiothorac Surg. 2014;45:1082–8. 24412832 10.1093/ejcts/ezt547

[14] Kume Y, Fujita T, Fukushima S, Shimahara Y, Matsumoto Y, Yamashita K, et al. Intracranial mycotic aneurysm is associated with cerebral bleeding post-valve surgery for infective endocarditis. Interact Cardiovasc Thorac Surg. 2018;27:635–41. 29701786 10.1093/icvts/ivy126

[15] Eishi K, Kawazoe K, Kuriyama Y, Kitoh Y, Kawashima Y, Omae T. Surgical management of infective endocarditis associated with cerebral complications. J Thorac Cardiovasc Surg. 1995;110:1745–55. 8523887 10.1016/S0022-5223(95)70038-2

[16] Sorabella RA, Han SM, Grbic M, Wu YS, Takayama H, Kurlansky P, et al. Early operation for endocarditis complicated by preoperative cerebral emboli is not associated with worsened outcomes. Ann Thorac Surg. 2015;100:501–8. 26116483 10.1016/j.athoracsur.2015.03.078 PMC4523437

[17] Fuse I, Higuchi W, Toba K, Aizawa Y. Inhibitory mechanism of human platelet aggregation by nafamostat mesilate. Platelets. 1999;10:212–8. 16801094 10.1080/09537109976040

[18] Murase M, Usui A, Tomita Y, Maeda M, Koyama T, Abe T. Nafamostat mesilate reduces blood loss during open heart surgery. Circulation. 1993;88:432–6. 8222190

[19] Sanfilippo F, Curro JM, La Via L, Dezio V, Martucci G, Brancati S, et al. Use of nafamostat mesilate for anticoagulation during extracorporeal membrane oxygenation: a systematic review. Artif Organs. 2022;46:2371–81. 35531906 10.1111/aor.14276

